# Impact of Marine Submergence and Season on Faunal Colonization and Decomposition of Pig Carcasses in the Salish Sea

**DOI:** 10.1371/journal.pone.0149107

**Published:** 2016-03-01

**Authors:** Gail S. Anderson, Lynne S. Bell

**Affiliations:** Centre for Forensic Research, School of Criminology, Simon Fraser University, Burnaby, British Columbia, Canada; Naturhistoriska riksmuseet, SWEDEN

## Abstract

Pig carcasses, as human proxies, were placed on the seabed at a depth of 300 m, in the Strait of Georgia and observed continuously by a remotely operated camera and instruments. Two carcasses were deployed in spring and two in fall utilizing Ocean Network Canada’s Victoria Experimental Network under the Sea (formerly VENUS) observatory. A trial experiment showed that bluntnose sixgill sharks could rapidly devour a carcass so a platform was designed which held two matched carcasses, one fully exposed, the other covered in a barred cage to protect it from sharks, while still allowing invertebrates and smaller vertebrates access. The carcasses were deployed under a frame which supported a video camera, and instruments which recorded oxygen, temperature, salinity, density, pressure, conductivity, sound speed and turbidity at per minute intervals. The spring exposed carcass was briefly fed upon by sharks, but they were inefficient feeders and lost interest after a few bites. Immediately after deployment, all carcasses, in both spring and fall, were very rapidly covered in vast numbers of lyssianassid amphipods. These skeletonized the carcasses by Day 3 in fall and Day 4 in spring. A dramatic, very localized drop in dissolved oxygen levels occurred in fall, exactly coinciding with the presence of the amphipods. Oxygen levels returned to normal once the amphipods dispersed. Either the physical presence of the amphipods or the sudden draw down of oxygen during their tenure, excluded other fauna. The amphipods fed from the inside out, removing the skin last. After the amphipods had receded, other fauna colonized such as spot shrimp and a few Dungeness crabs but by this time, all soft tissue had been removed. The amphipod activity caused major bioturbation in the local area and possible oxygen depletion. The spring deployment carcasses became covered in silt and a black film formed on them and on the silt above them whereas the fall bones remained uncovered and hence continued to be attractive to large numbers of spot shrimp. The carcass remains were recovered after 166 and 134 days respectively for further study.

## Introduction

The fate of a human body in water is not well understood. A great deal of research has been conducted on carcass decomposition and insect colonization in a variety of terrestrial habitats worldwide [1–3] yet little work has been performed in the ocean. The inaccessibility of the marine environment makes such work challenging, yet the large number of commercial accidents, recreational deaths and body depositions which occur in the ocean, make it an important area of research concerning both the ecology of death itself, and of concern here, forensic investigations of deaths in the ocean.

Large carcass falls such as those of whales [4–10], sharks [11] and porpoises [12], as well as those of large extinct animals [13–15] and even invertebrates [16, 17] have been studied but many of these aquatic mammals decompose in a manner very different from that of humans or human-sized mammals. Large carcasses such as whales go through four major decompositional stages over a period of years, and complete decomposition can take decades [4]. Therefore, much of our knowledge of human decomposition in the ocean comes from anecdotal reports of individual body recoveries [18–22].

In 2000, studies were conducted in the Howe Sound region of the Salish Sea (the waters between Vancouver Island, mainland BC and Washington), comparing pig (*Sus scrofa* L., 1758) decomposition and faunal colonization in spring and fall at two depths [23]. Although very valuable data on taphonomic changes, scavenging, and impact of depth and season were generated, these experiments utilized divers to document the carcasses over time, which greatly limited the number of observations possible.

Those experiments were expanded into work in the Saanich Inlet in collaboration with the Victoria Experimental Network Underseas (VENUS, now Oceans Network Canada (ONC)) [24]. This is an underwater cabled laboratory in the Salish Sea which allows researchers to conduct experiments in several undersea habitats in real-time using remotely operated equipment, greatly expanding the amount and quality of data that could be collected.

The research reported here describes the next in an ongoing series of experiments with the VENUS laboratory, utilizing a range of chemical sensors and instruments as well as a camera programmed to record carcasses underwater every 15 minutes. These experiments were conducted in the Strait of Georgia, between the mainland of southern British Columbia and Vancouver Island using pig carcasses as human proxies. The objectives of this study were to understand the impacts of biotic and abiotic parameters on human proxies in a deep coastal marine environment in order to apply such knowledge into both an ecologic and forensic context. The impacts of a number of biotic and abiotic parameters on carcasses over a six month period were compared and contrasted in two seasons, spring and fall.

## Materials and Methods

### Ethics Statement

Simon Fraser University Animal Care Committee permission was obtained to purchase dead pigs, Animal Care Permission #1027CR-11. The field studies did not involve endangered or protected species. Pigs were euthanized with a humane pin-gun or by electrocution by a licensed butcher and were received after death. No live vertebrates were involved. Carcasses were placed in the ocean. No specific permission required. GPS coordinates were 49°02.4023', -123°25.5292’ and 49°02.3845', -123°25.5610’.

### Research Site

The Salish Sea encompasses the coastal waters from the south coast of British Columbia to the northwestern tip of Washington State and includes the Strait of Georgia, Juan de Fuca Strait and Puget Sound. The research site was in the Strait of Georgia, a deep basin between Vancouver Island and mainland British Colombia [25].

### Ocean Network Canada’s VENUS Observatory and Instruments

The Victoria Experimental Network Underseas (VENUS) is an Ocean Networks Canada cabled underwater observatory. It is based out of the University of Victoria on Vancouver Island, British Columbia and delivers real-time, high speed data to researchers globally [25].

A digital webcam (AXIS Q6034, 720p HD) was positioned directly above the carcasses with four lighting arrays. It was powered on continuously although lights only came on every 15 minutes for a period of 2 minutes, during which time the camera was programmed to pan both carcasses and the surrounding area. The camera could also be operated manually for close-up images. In the spring experiment, the carcass and camera platform was directly connected to a VENUS Instrument Platform (VIP-08, 49°02.3845' -123°25.5610’, depth 300m) which supported several instruments. A SeaBird CTD (16 Plus 6936) recorded temperature (°C), salinity, (psu), pressure (decibar), conductivity (S/m), density (kg/m^3^), Sigma T ((kg/m^3^) and sound speed (m/s) every 60 seconds, a Wetlabs NTU (ECO-NTU(RT) 319 recorded turbidity (nephelometric turbidity units (NTU)) every 60 seconds and an oxygen optode (Aanderaa Optode 4175 S/N 0581) recorded dissolved oxygen levels (mL/L) [25].

In the fall experiment, a new, separate camera and carcass platform was designed and was separated from the VENUS instrument platform by 69 m (VIP-11, Central Strait of Georgia, 49°02.4143' -123°25.5826’, depth 299m). The instruments were mounted on the VIP as before but duplicate instruments were also mounted on the carcass platform (SG-HDW-01, Central Strait of Georgia, 49°02.4023' -123°25.5292’, depth 297 m). The instruments included a CTD (SeaBird 16 Plus 4998), a wetlabs NTU (ECO-NTUS-461) and an oxygen optode (Aanderaa Optode 4175C S/N 1794).

### Carcass Deployment

**Fig 1:**
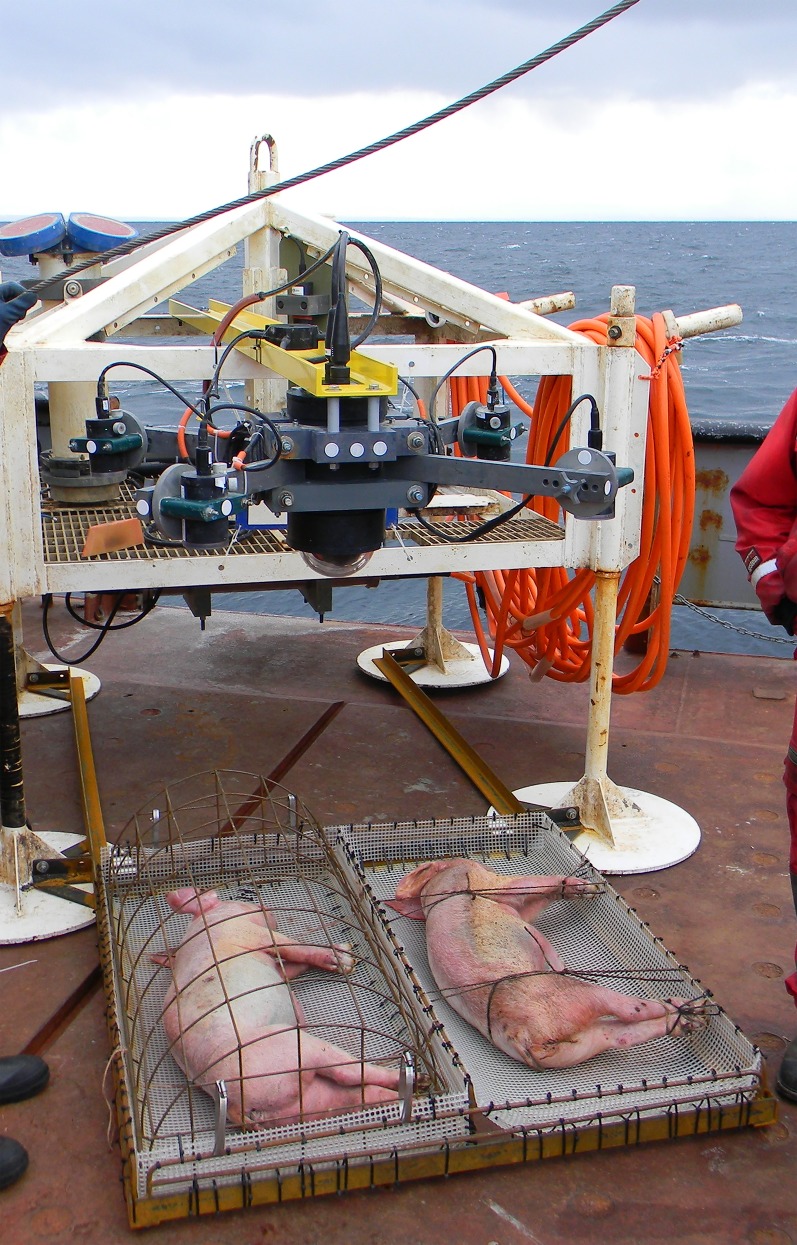
The carcass platform with instruments mounted and carcasses tied in position before deployment. Cage, trays, frame and camera system frame designed by Chris Sundstom, VENUS Instrument Platform (VIP) designed by Paul Macoun (Ocean Network Canada’s VENUS observatory).

In spring (February) 2010 a single, freshly killed pig carcass (~23 kg) was deployed directly on the ocean floor at a depth of 300 m, under a camera mounted on a tripod. It was weighted to prevent removal from camera range but was otherwise openly exposed in the same manner as carcasses had been in previous experiments in the nearby waters of Saanich Inlet [24]. The carcass immediately attracted lyssianassid amphipods (Family Lyssianassidae) and other zooplankton, including shrimp in the Order Mysida. Unfortunately, within 8 hours, *Hexanchus griseus* (Bonnaterre, 1788) the bluntnose sixgill shark was attracted and fed upon the carcass. Very shortly after the first shark began to feed, several other sharks were attracted and the carcass was rapidly devoured (S1 Video). Therefore, a new experimental protocol was designed (Fig 1).

The new design involved a platform (designed by Chris Sundstom, ONC) which exposed one carcass completely, with the hope that it might not be entirely consumed, but protected a second, adjacent carcass with a metal frame with large gaps (14-15cm x10-12 cm) which would prevent shark feeding but not impede access to invertebrates or most fish (Fig 1). The base of the platform was made of plastic 1 cm mesh which allowed the carcasses to rest on the sea bed but also facilitated skeletal recovery at the end of the experiment for further study. The carcass platform itself was attached to an instrument platform (designed by Paul Macoun, ONC) and the digital video camera was positioned directly above the carcasses (Fig 1).

Two freshly euthanized domestic pig carcasses (~24 kg) were deployed in spring, on 26 February 2012, and a further two (~21 kg) were deployed in fall, on 24 October 2013. In each deployment, the carcasses were tied onto the frame of the carcass platform, one exposed and one under the cage bars.

The carcass platform was lowered from the research vessel into the water and then positioned at the site underwater by a remotely operated vehicle. The vehicle then connected the instruments and camera on the carcass platform to the NODE for data transmission. The day of submergence was recorded as Day 0. Once the carcasses were deployed, they were not physically accessed until the skeletal elements were recovered approximately six months after placement.

### Data Collection

Once deployed, the camera and lights were programmed for the lights to come on every 15 minutes for approximately two minutes, during which time the camera would pan the two carcasses, spending several seconds recording head, torso and rear of each carcass as well as the surrounding area. These 96 videos a day were then downloaded from the VENUS website (www.oceannetworks.ca) for observation and analysis. At first, every video was watched, then viewing was reduced to hourly unless major changes occurred between hours, then the intermediate videos would be observed. Once the carcasses were skeletonized, observations were reduced to every four hours and finally to daily, although if any changes were observed, the previous videos were reviewed. All other instruments recorded data every 60 seconds and these data were downloaded from the VENUS website for analysis. All data and videos can be accessed at www.oceannetworks.ca, under Data and Tools, Data Download Tools

The remains of the carcasses were recovered on Day 166 in the spring experiment (10 August 2012) and Day 134 in the fall experiment (7 March 2014) by a remotely operated vehicle which placed a pre-designed Plexiglas lid lined with foam over the exposed carcass bones to protect them as the platform was raised up through the water column. All bones were recovered for immediate examination and later study. During the spring remains recovery cruise, baited traps were deployed for a few hours to collect amphipod samples.

## Results

### Carcass Scavenging

Lyssianassid amphipods were attracted immediately to carcasses in both spring and fall, beginning to settle on the carcasses within a few hours of submergence. Baited traps collected amphipods in fall and these were identified to the *Orchomene* complex, *Orchomenella* aff. *obtusa* (Sars, 1891). However, this complex is in taxonomic flux at this time, so a specific identification is difficult.

In spring, a bluntnose sixgill shark swam over the carcasses a few hours after deployment but was unable to access the caged carcass (S2 Video, Table 1). It bit the exposed carcass in the abdominal area, causing damage but did not remove much tissue. Several sharks were subsequently attracted and some bit the carcass, disturbing roosting amphipods. The sharks did not bite the carcasses after 24 h although still swam over the carcasses until they lost interest 48 h after submergence, by which time both carcasses were covered in thick layers of amphipods. No sharks fed on the carcasses in fall, although shadows swimming over the carcasses in the first 24 h suggested that sharks did show an interest.

**Table 1:** Presence or absence of the major fauna on pig carcasses in fall and spring in Strait of Georgia at 300m. F = fall, Sp = spring, Relative abundance for most species could only be estimated but is given as approximately x = 1–5, xx = 5–25, xxx = 25–100, xxxx = 100–1000, xxxxx = many 1000s.

Day(s) after Submergence	Amphipods		*Metacarcinus magister*		*Pandalus platyceros*		*Hexanchus griseus*		*Eualus* sp.		*Sebastolobus alascanus*		*Heptacarpus cf*. *tenuissimus*		*Other crabs*	
	Sp	F	Sp	F	Sp	F	Sp	F	Sp	F	Sp	F	Sp	F	Sp	F
**0**	xxxx	xxxx			x		x									
**1**	xxxxx	xxxxx		x	xx		x									
**2**	xxxxx	xxxxx			xxx		x									
**3**	xxxxx	xxxxx			x									x		
**4**	xxxxx	xxxxx														
**5**	xxxx	xxxx			x									x		
**6–10**	xxxx	xxxx	x		xxx	x				xx	x	x		x		x
**11–17**	xxx		x	x	x	xxx				xxx	x			x		x
**18–22**	xx			x	x	xx				xxx	x					x
**23–27**			x	x	x	xxx				xxx	x					
**28–32**			x		xx	xxx				xx				xx	x	
**33–37**	x		x		xx	xxxx				xx	x			xx	x	x
**38–50**			x	x	xxx	xxxx					x			x	x	
**51–60**			x	x	xx	xxxx								x	x	
**61–70**				x	x	xxxx					x			x	x	x
**71–80**			x		x	xxxx					x				x	
**81–90**			x		x	xxxx								x	x	x
**91–100**			x		x	xxx										
**100-**			x		x	xxx					x			x	x	

**Fig 2:**
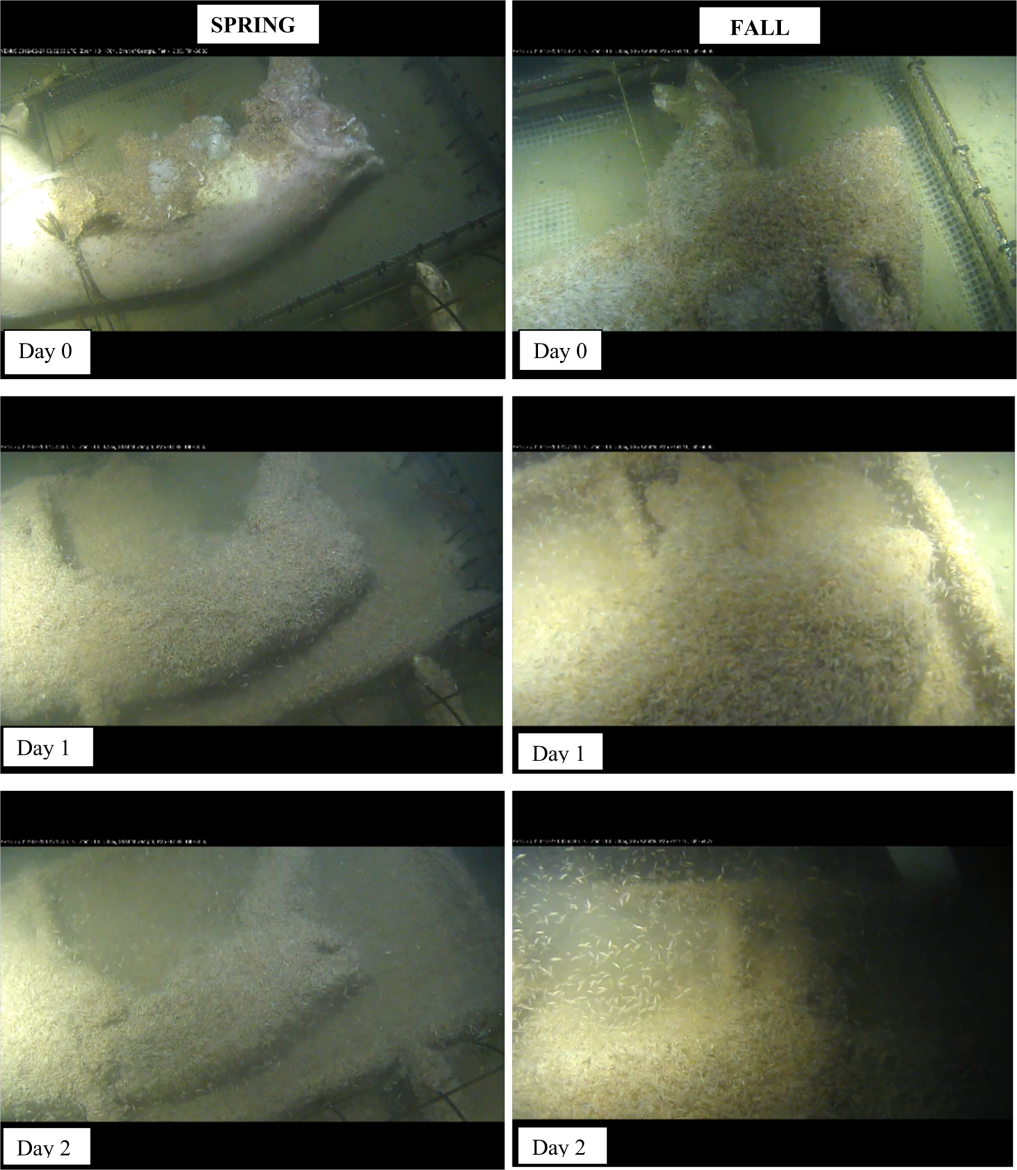
*Amphipods (*Orchomenella *aff*. obtusa (Sars, 1891), *Lyssianassidae) colonization of carcasses*, *Days 0–2 after deployment*, *spring and fall (Ocean Network Canada’s VENUS observatory)*.

Lyssianassid amphipods were present on the carcass in increasing numbers over Days 1–4, roosting in thick layers on the carcasses and numbering in the thousands or more (Fig 2, Table 1). Cut areas created by sharks were attractive but all the skin of both carcasses was covered by Day 1 with amphipods covering not only the carcasses but also the surrounding cage area by Day 1 in fall and Day 2 in spring, extending from the platform by at least one metre (Fig 2).

On several occasions, something, probably a fish, caused the amphipods to be sloughed off an area of a carcass exposing intact skin indicating that they were entering the carcass itself and preferentially feeing on internal organs and tissue rather than skin, with the skin being the last to be consumed. When areas of skin were exposed, it was rapidly recolonized by amphipods. Fourteen hours after submergence in fall, amphipods were sloughed off the facial area of the exposed carcass, however, and damage to the face and ear was apparent suggesting they were feeding at the orifices. The amphipods appeared to be moving frenetically on the carcasses but close up videos revealed that only the upper layers were moving with those close to the carcass or substrate fairly immobile. The numbers of amphipods continued to increase until the middle of Day 4 in spring and Day 3 in fall, when all carcasses were skeletonized (Table 1). After skeletonization, the amphipods receded rapidly (Table 1). Small groups of amphipods remained on the cage floor until Day 7 in spring and Day 9 in fall. Once the amphipods receded, 50–100 heart urchin tests (Order Spatangoida) could be seen under and surrounding the platform area. These may have been exposed by bioturbation caused by the amphipods. On Day 16, amphipods returned to the spring carcass bones giving them a furred appearance. They remained in low numbers for a few days then dispersed.

**Fig 3:**
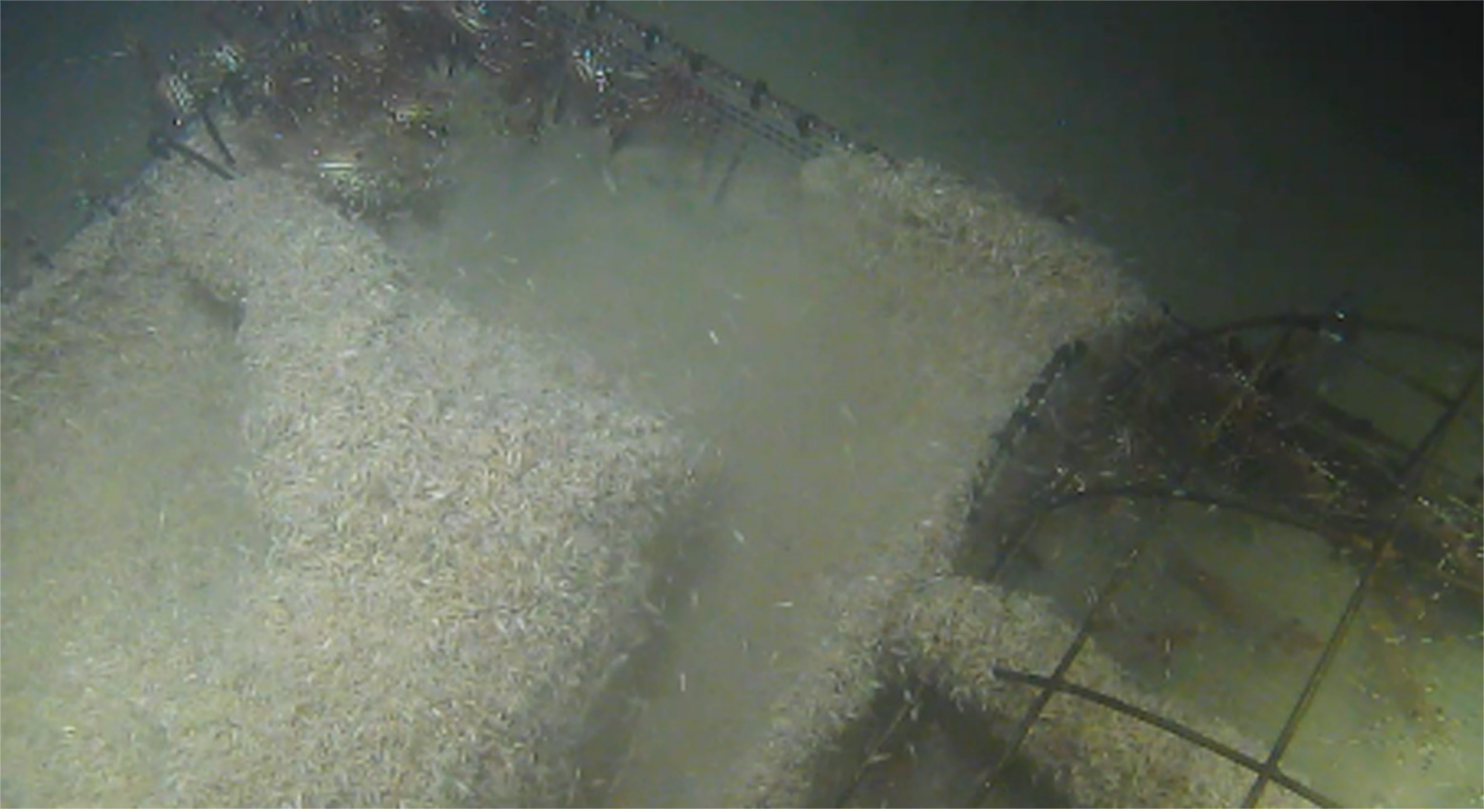
Pandalus platyceros *Brandt*, *1851 (Three Spot Shrimp) being excluded from the carcass by encroaching layers of lyssianassid amphipods*. Day 2, spring deployment (Ocean Network Canada’s VENUS observatory).

In the first few days of submergence (Days 0–2) a few spot shrimp, *Pandalus platyceros* Brandt, 1851, were present in spring only (Table 1) and appeared to actively avoid the amphipods, only very rarely being seen feeding in amphipod-covered tissue. As the amphipod numbers increased, *P*. *platyceros* were restricted to one corner of the cage, then were occluded entirely by Day 3 (Fig 3).

**Fig 4:**
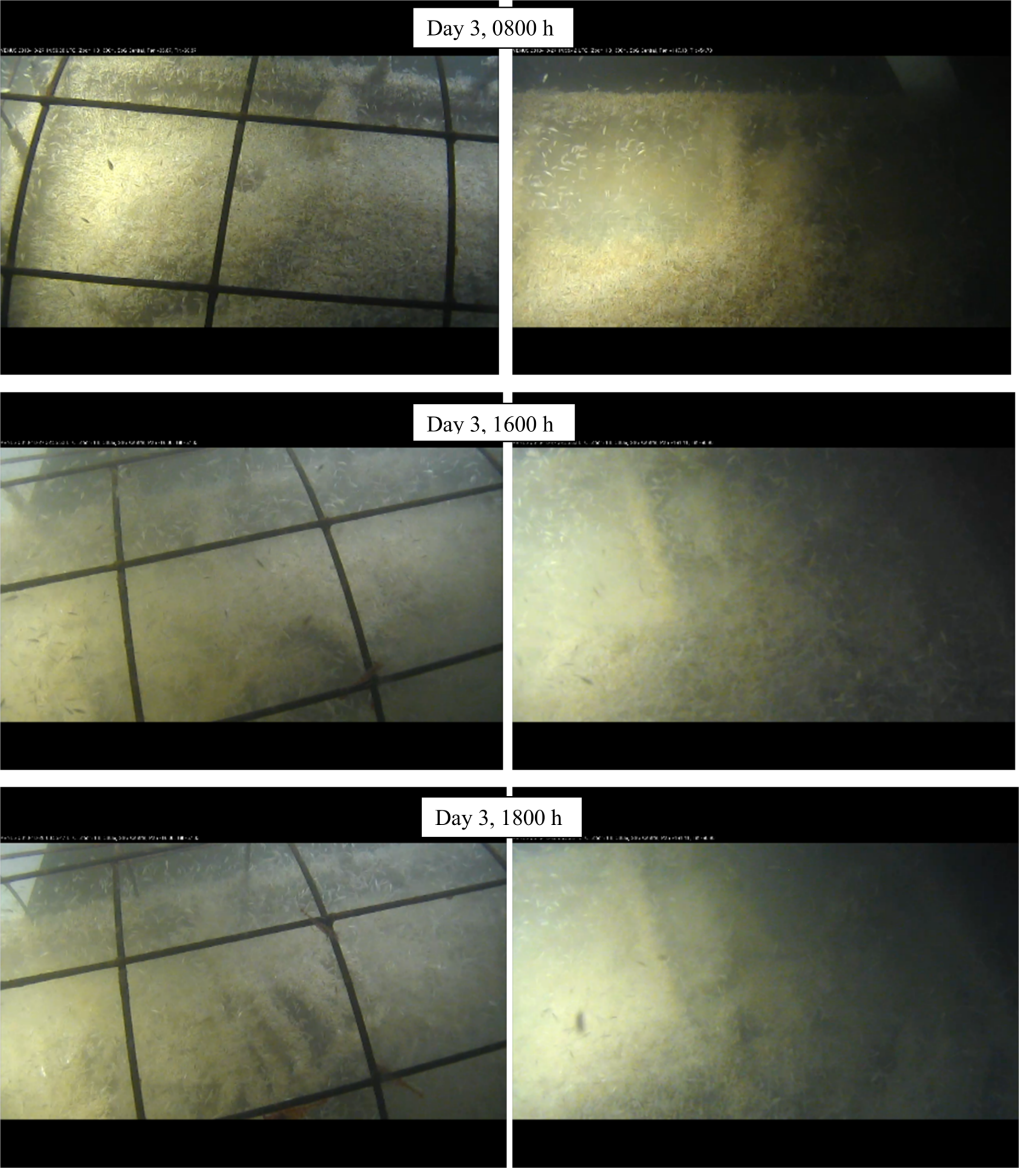
Progression of skeletonization in fall carcasses, Day 3 (Ocean Network Canada’s VENUS observatory).

Although covered in amphipods, carcass bulk could be approximated and could be seen to begin depleting by Day 2, starting at the rump and neck areas. By the end of Day 3 the rear legs of the spring exposed carcass were skeletonized with tissue loss along the spinal region and abdominal areas in both carcasses and by this time in fall, the entire carcasses were skeletonized (Fig 4).

**Fig 5:**
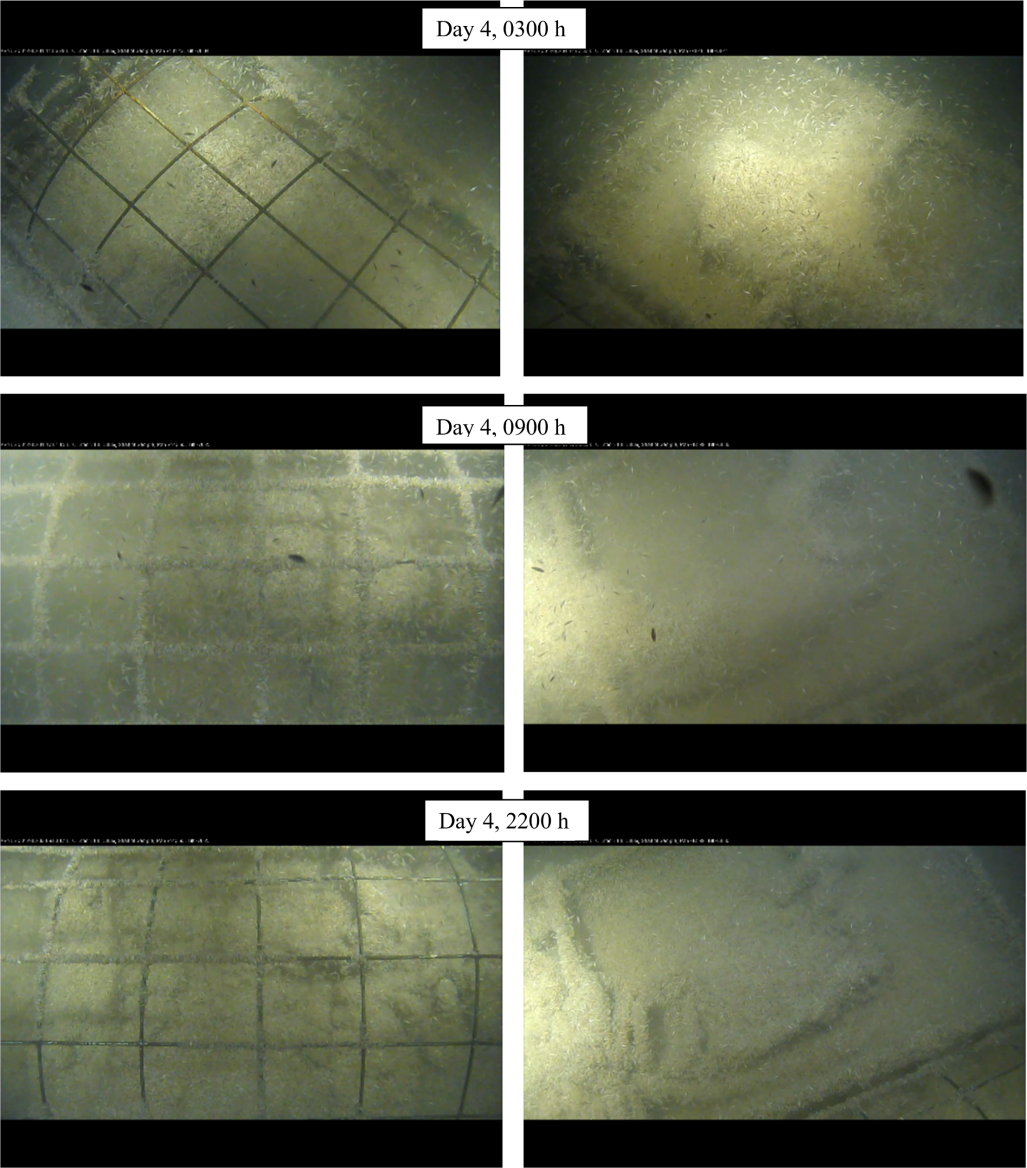
Progression of skeletonization and movement of amphipods on cage in spring carcasses, Day 4 (Ocean Network Canada’s VENUS observatory).

In spring, amphipods covered not only the carcasses and substrate but also covered the entire cage bars, encroaching on the bars at the beginning of Day 4, covering all the bars by midday and then beginning to recede by 1900 h with only a few remaining by 2100 h, exposing the skeletonized carcass (Fig 5). Despite the damage caused by sharks to the exposed carcass, both were skeletonized at the same time. In both seasons, the actions of the amphipods alone moved the skeletal elements out of anatomical position.

Once the amphipods had skeletonized the carcasses, the bones began to be covered in a fine silt and many *P*. *platyceros* returned to the spring carcasses and appeared for the first time on fall carcasses. In spring, large numbers of *P*. *platyceros* consumed the cartilage on the bones by Day 10 and further moved the skeletal elements and heart urchin tests. On Day 9, a giant pacific octopus (*Enteroctopus dofleini* (Wülker, 1910)) approached the spring caged carcass and explored the cage and contents, disturbing very little but driving away any fauna present (S3 Video). In fall, a few *P*. *platyceros* were present but many small red shrimp (*Eualus* sp.) picked at the cartilage, bones and substrate.

**Fig 6:**
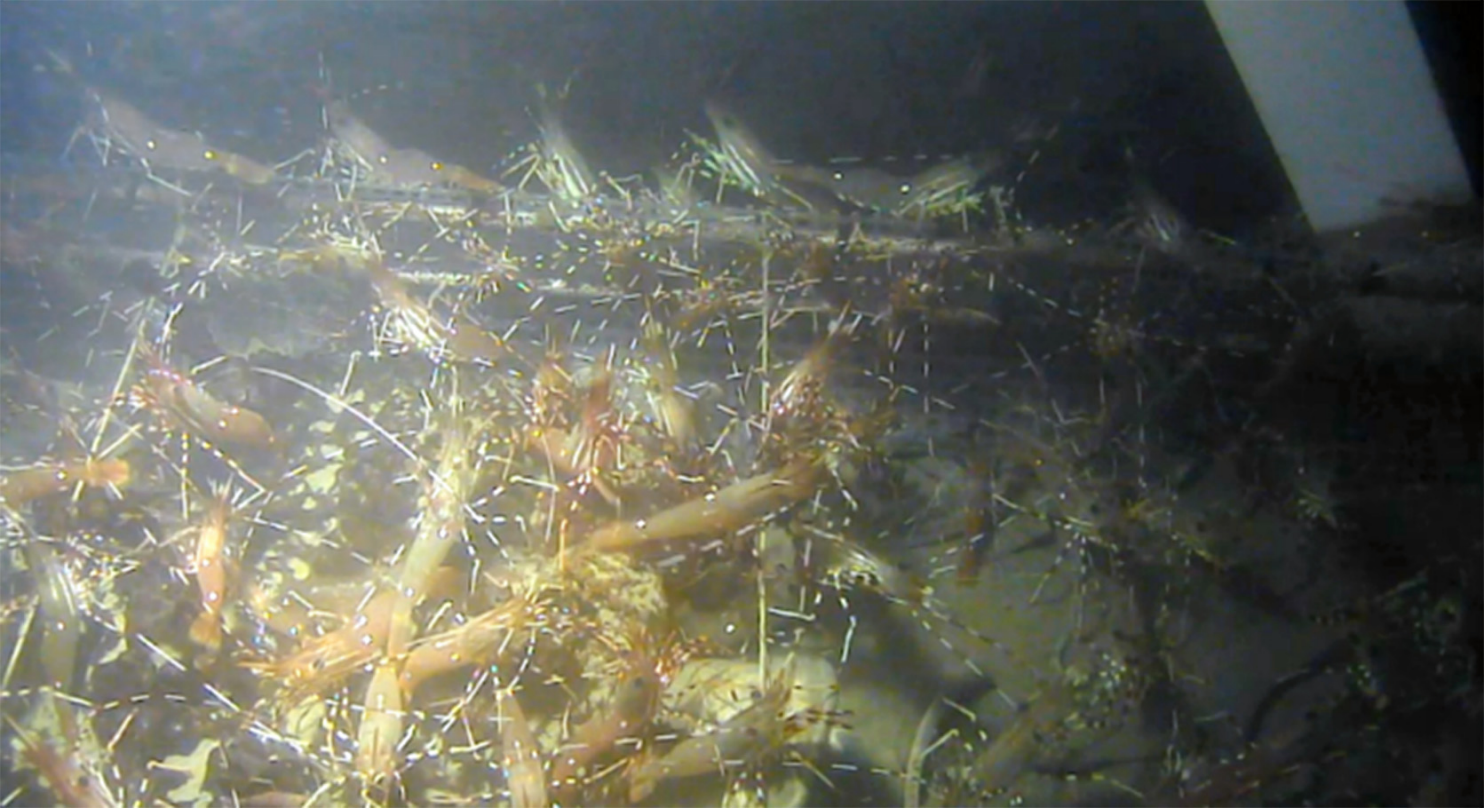
*Hundreds of* Pandalus platyceros *Brandt*, *1851*, *on the bones in fall*, *Day 42 (Ocean Network Canada’s VENUS observatory)*

After Day 10, *P*. *platyceros* were seen occasionally on spring carcasses in low numbers (1–11) but in fall, numbers of both adult and juvenile *P*. *platyceros*, together with other shrimp such as *Eualus* sp. and *Heptacarpus cf*. *tenuissimus* Holmes 1900, increased until over 100 specimens were present on the bones, and numbers remained very high until recovery (Fig 6).

After this, aside from the large numbers of shrimp almost continuously on the fall carcass bones, only a few fauna were present on either set of carcasses. On occasions (Table 1), a few individual *Metacarcinus magister* (Dana, 1852), (Dungeness crabs), were observed resting in the platform area with the carcasses and sometimes picking at the bones. The crabs would sometimes pick up bones and moved the crania on several occasions. Hermit crabs (Order Decapoda, superfamily Paguroidea), decorator crabs (Order Decapoda, Superfamily Majoidea) and a tanner crab (*Chionoecetes tanneri* Rathbun, 1893) were occasionally present. In fall on Day 68, a giant pacific octopus, *E*. *doflieni*, was first observed investigating the platform and it remained close to the carcasses, mostly on top of the cage bars, for three days. Unlike the earlier specimen seen in spring, this animal moved the bones around inside the cage. All fauna, including the large number of *P*. *platyceros*, were driven away and did not return until three days after the octopus was last observed. Once the amphipods had left, from Day 11 onwards, one to three Shortspine thornyhead fish (*Sebastolobus alascanus* Bean, 1890) frequently swam over the platform and through the cage bars, primarily in spring, often resting in the cage for many hours at a time (Table 1). They did not appear to feed on the bones or fauna but merely rested in the cage, as perhaps it provided shelter.

**Fig 7:**
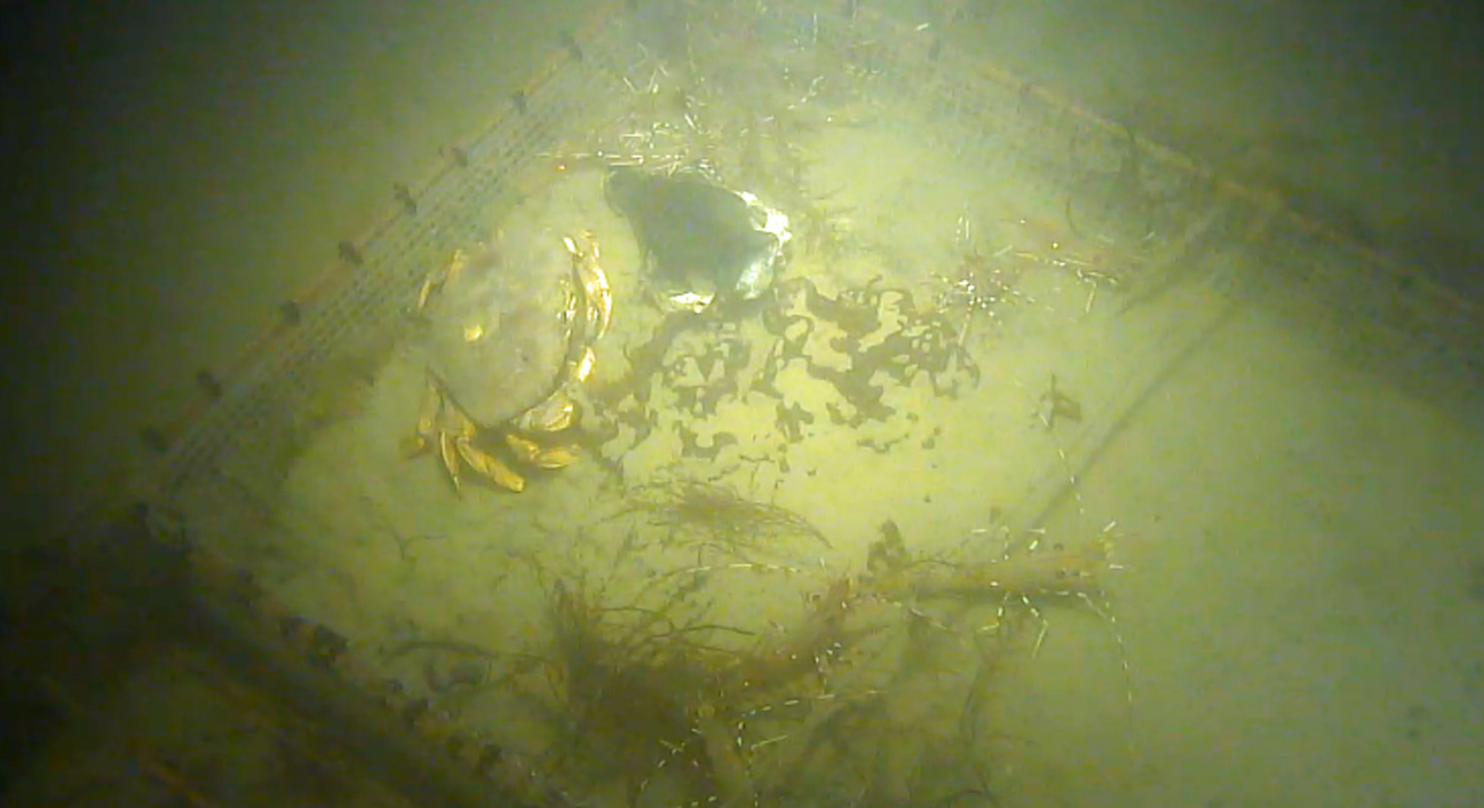
*Cranium moved by Dungeness crab (*Metacarcinus magister *(Dana*, *1852)) shows dark coloration on area previously protected by silt*. Day 100, spring deployment (Ocean Network Canada’s VENUS observatory).

After skeletonization, the bones were progressively covered in silt and a black coloration began to form on the bones and the surrounding silt, beginning on Day 26 in spring and Days 23 and 24 in fall. This film increased over time, spreading further into the surrounding silt. It gradually developed a greyish hue. In spring, the carcasses became covered in silt, with the postcranial bones becoming completely buried, whereas only a light silt covering developed in fall. When a large crab (*M*. *magister)* turned the cranium over in spring, the newly exposed area was solid black, which turned to grey within a few hours (Fig 7).

### Physical and Chemical Measurements

**Fig 8:**
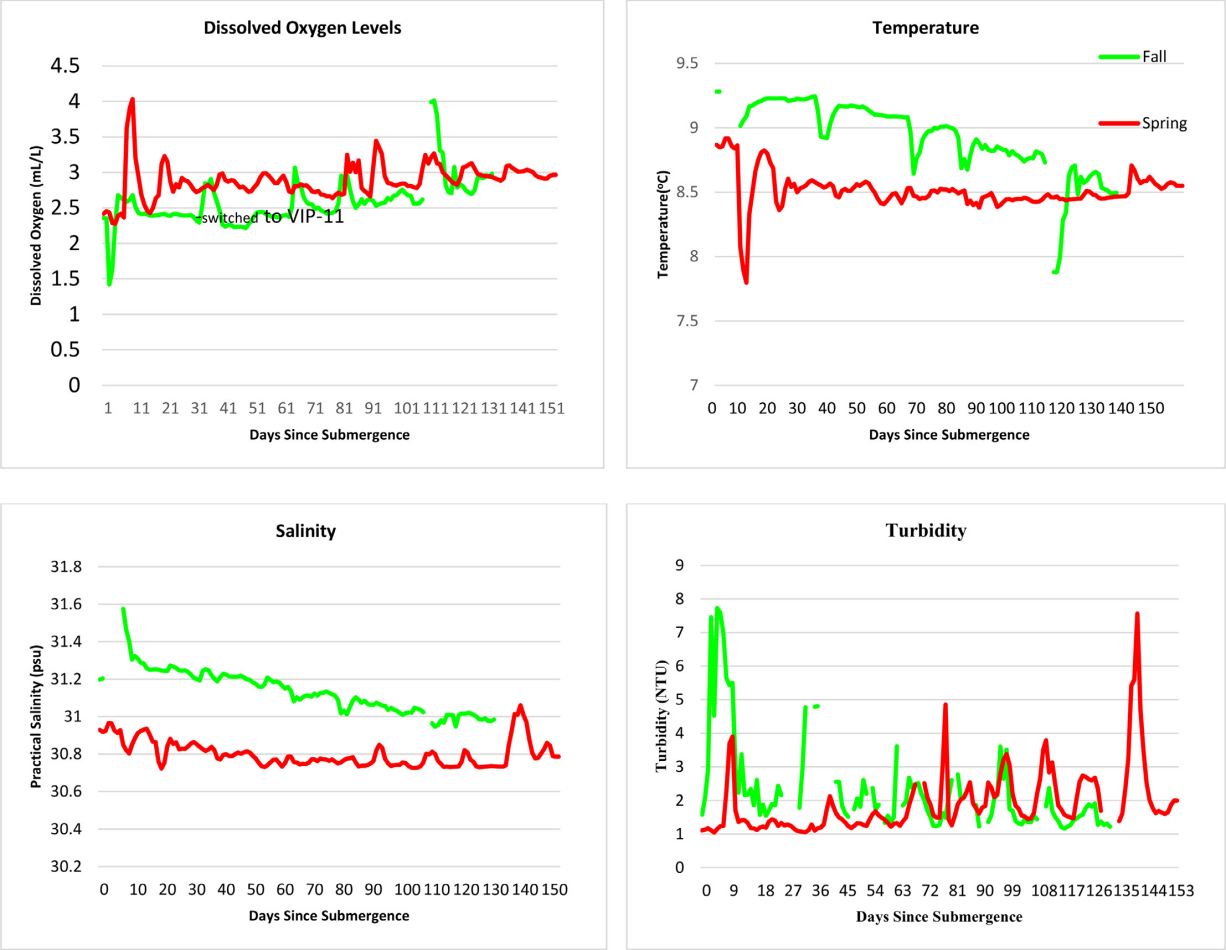
Daily averages of dissolved oxygen levels (mL/L), temperature (°C), salinity (psu), and turbidity (NTU) at a depth of 300 m in the Strait of Georgia in spring and fall (Ocean Network Canada’s VENUS observatory).

Dissolved oxygen, temperature, salinity, and turbidity were measured (Fig 8).

In spring from Days 9–11, a major physical oceanographic event was recorded. Fresh, cold water, probably resulting from rain and river run-off originating from near the water surface, was recorded passing through the site and probably moving to deeper areas [26]. This impacted all the physical and chemical measurements (Fig 8). The cold, fresh water lowered salinity, and temperature, increased density and increased oxygen levels. It also greatly increased the silt in the area, making it difficult to see the carcasses. During this time, fewer fauna were observed, although at the start of this period, an octopus was not deterred.

**Fig 9:**
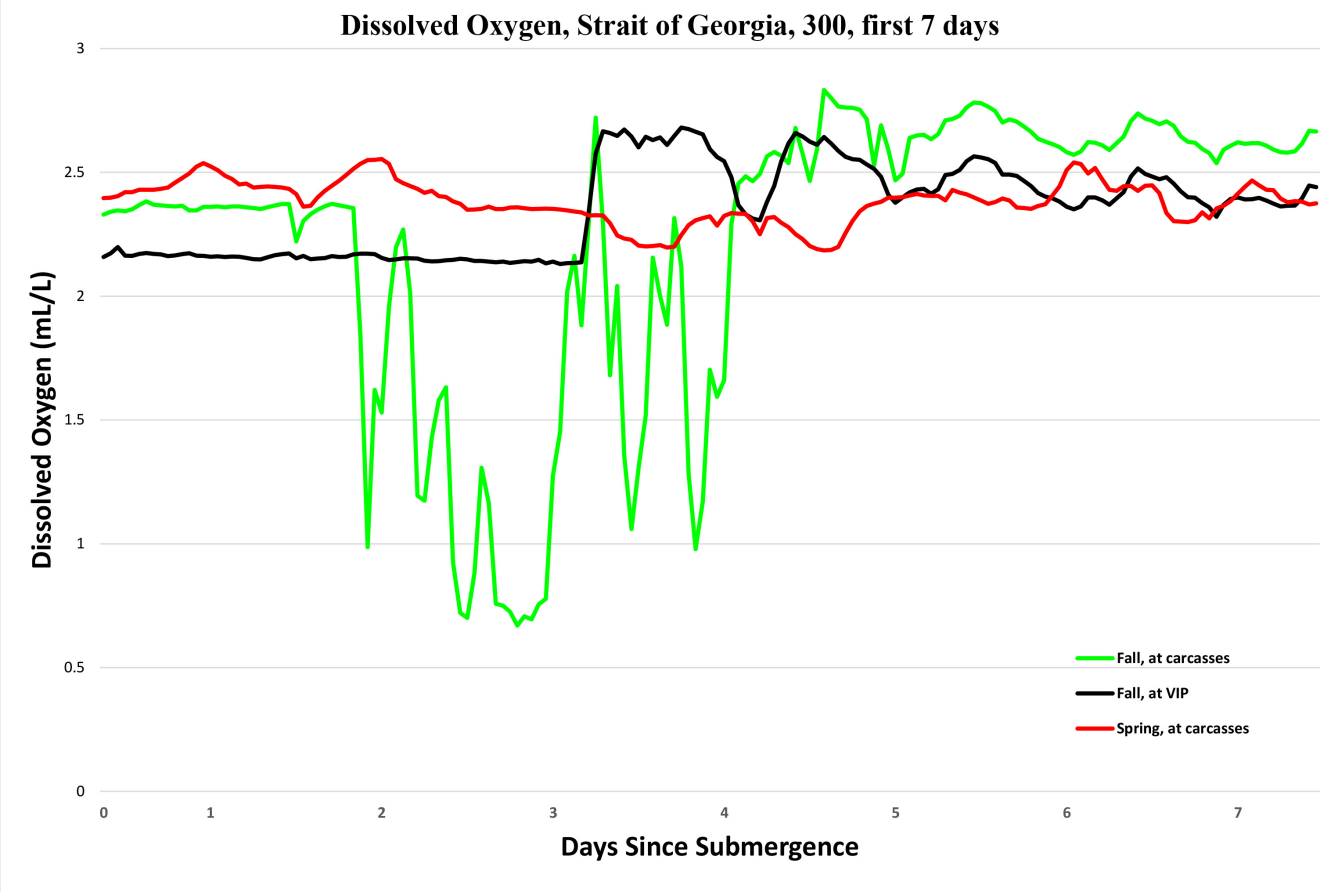
Hourly dissolved oxygen levels (mL/L) at a depth of 300 m in the Strait of Georgia in spring and fall for the first 7 days, taken from Aanderaa optodes mounted on VIP-08, the carcass platform in spring and the carcass platform and VIP-11 in fall (Ocean Network Canada’s VENUS observatory).

As expected, oxygen levels were slightly higher overall in spring than fall, and Fig 8 shows the sudden increase in oxygen caused by the oceanographic event. However, there is also a very marked drop in oxygen levels shortly after deployment in fall. This can be seen more clearly in Fig 9, which shows the hourly averages of dissolved oxygen levels for the first seven days at VIP-08 in spring, and at both VIP-11 and the carcass platform in fall.

This dramatic drop in oxygen did not occur at the VIP, 69 m distant, showing that it was not a wide ranging event such as that seen when the oxygen increased dramatically during the fresh water run-off in Days 9–11 in spring. It also coincides exactly with the majority of the amphipod activity in fall, beginning two days after submergence and returning to normal once the amphipods receded. It is possible this dramatic drop in oxygen levels is a drawdown resulting from the frenetic metabolic activity of the amphipods. A similar drop in dissolved oxygen, however, was not observed during the amphipod activity in spring. This may be due to the locations of the optodes. In spring, the VIP and the carcass platform were combined, and all instruments were on the structure. The carcass and camera part of the platform, however, was in front of the VIP. This meant that the optode was approximately 1.5 m above the ground and a further 1.5 m distant from the carcasses, making it 2.25 m from the carcasses. In fall, the camera and carcass platform was separated from the VIP by 69 m and the carcass platform was directly beneath the structure. An optode additional to the VIP optode was placed at carcass level, approximately 30 cm or less from the caged carcass. This suggests that, if the actions of the amphipods were capable of depleting the water of oxygen, then this is a very local phenomenon.

Temperature was similar for both deployments with fall being slightly warmer for the duration (Fig 8). The impact of the sudden cold-water influx is clear, resulting in a rapid drop of more than 1^°^C for two days, then a return to normal. Salinity was slightly higher during the fall deployment.

## Discussion

### Scavenger Activity

All carcasses were immediately attractive to lyssianassid amphipods, including the preliminary carcass which was rapidly consumed by bluntnose sixgill sharks. Sharks fed briefly on the spring exposed carcass and caused large open wounds, but were inefficient feeders and did not remove much soft tissue. The bluntnose sixgill shark is a wide ranging demersal deep water species feeding on a range of prey species and carrion [27, 28]. It is usually found in deep waters, coming to shallower waters at night to feed, although in these experiments as well as the preliminary carcass deployment, it fed primarily during the daytime hours [29].

Carcasses were rapidly covered in layers of amphipods which varied in thickness (~4 cm on carcasses and surrounding areas, 2–4 cm on cage edge and 1 cm on cage bars). Once covered, sharks and all other invertebrates no longer appeared attracted to the carcasses. Although shadows of sharks passed over the fall carcasses, none fed. One shark bit into the exposed spring carcass after amphipods had settled, taking tissue and many amphipods into the mouth, then expelling amphipods through the gill slits and leaving. It is possible that the amphipods proved irritating, driving the sharks away. Lyssianassid amphipods are attracted to carrion by chemosensory stimulation, although mechanical stimulation caused by the carcass reaching the bottom and the presence of the ROV may also have played a factor [30]. Hydro-acoustic stimuli from a carcass fall has been shown to attract resting scavengers from several hundred meters distance [31, 32]. However, the continued attraction to the carcass over several days indicates that chemosensory attraction is most important [33].

The amphipods were many animals deep, with carcasses covered in layers of amphipods at least 4 cm deep on the carcasses themselves and although on the cage bars seemed to be only one animal deep, they appeared to be up to 2–4 cm deep when roosting on the cage edge and on the substrate surrounding the cages at times. This is similar to that described by recovery divers on drowning victims where entire bodies, including full dive suit, and mask, are covered in amphipods several animals deep [22]. Other studies have shown that it is common for lyssianassid amphipods to cover carrion in large numbers, even when the carrion is extremely small, for example, images of the accidental observation of a dead bathypelagic shrimp, *Pasiphaea tarda* Krøyer, 1845 (31 mm in carapace length) at 5,551 m in Fram Strait in the Arctic ocean showed that it was deeply covered in hundreds of a *Uristes* sp. of scavenging amphipod which was new to science [34].

When amphipods were sloughed off the carcasses on occasions, the skin could be seen to be intact, so the amphipods were clearly entering the carcasses, through wounds and orifices and preferentially feeding on muscle or organ tissue, eating the skin last. In baited experiments, amphipods have been shown to differentiate between tastes and textures of food, selectively feeding on fat and muscle tissue, leaving skin and connective tissue whole [35]. In experiments using whole or partial dolphin and porpoise carcasses in the north-east Atlantic, amphipods entered the carcasses through orifices or incisions, or simply burrowed through skin to reach muscle tissue. Amphipods breached the skin by 36 h, had preferentially removed muscle tissue over connective tissue, blubber and skin by 152 h and skeletonized the carcass within six days, although they did not eat the skin [36]. Considering the fact that these were larger carcasses than those deployed here, this is very similar to the amphipods progression observed, although here the skin was eventually consumed. Despite sharks opening several large areas of skin and removing some tissue in the exposed carcass, the two spring carcasses were skeletonized at exactly the same time, showing that the amphipods were easily able to penetrate undamaged skin.

In the above dolphin/porpoise experiments, funnel traps placed near the carcasses showed that there was a succession of eight amphipod species with numbers changing over time. In the present experiments, the amphipods were only observed, not trapped, except for a single baiting event that took place when the spring carcasses were recovered which collected species in the *Orchomene* spp. complex. Although the amphipods appeared to be uniformly similar during the skeletonization on camera, it is possible that more than one species was present. Once skeletonized, the sparser amphipods attending the carcasses may have been a different species. These carcasses were all deployed during daytime, at 0845 h local time in spring and 1310 h local time in fall, and all immediately attracted amphipods. Their activity on the carcasses continued unabated until the soft tissue had been entirely removed, with no observable diel change, although some *Orchomene* group amphipods have been shown to be only active nocturnally [37–39]. This was not true in this case.

The dramatic drop in oxygen during the mass amphipod feeding on the fall carcasses is extremely interesting. The lack of a similar drop in spring, despite equally frenetic amphipod behavior, may be due to the optode being positioned much further from the carcasses in spring. It is possible however, that it is an artefact, which could have been caused by the optode’s optical sensor being blocked with amphipods. Unfortunately, the videos of the experiment do not cover the optode area, so this cannot be ascertained but, in spring, although many amphipods covered the entire caged area including completely covering the bars of the cage briefly, this did not happen in fall, with the amphipods only coming up the bars slightly, suggesting it is unlikely they would have covered the optode. If the oxygen drop was genuine, it may have been low oxygen that repelled other fauna such as *P*. *platyceros*. Low oxygen levels would not have impacted the amphipods as *O*. *obtusa* can survive 10–33 h of anoxia at *in situ* conditions [40]. Respiration rates of two *Orchomene* spp. in response to bait odor were measured and indicated an elevated rate of oxygen consumption for up to eight hours [41], suggesting that the lowered oxygen levels may indeed be due to localized amphipod activity. This is an area that will be further explored in a future experiment.

### Decomposition

These carcasses did not exhibit any of the typical decompositional stages observed in bodies in fresh [42–44] or salt water [19, 20, 22] but instead, were rapidly consumed by scavengers, primarily the lyssianassid amphipods. In earlier experiments in Saanich Inlet, the carcasses were also consumed rather than decomposing, although not nearly as rapidly due to the much lower numbers of lyssianassid amphipods. However, in shallower waters in Howe Sound, distinct fresh, bloat, active, advanced and remains stages were observed as well as other characteristics often noted in aquatic environments [23]. This is similar to that reported from the Puget Sound area of the Salish Sea, just slightly south of the present experiments [21] and those in Monterey Bay in California [19], with soft tissue remaining for some time and being primarily lost due to decomposition and abrasion. Even in the warmer waters of the Tyrrhenian Sea in the south west Calabria region of Italy, a body washed ashore after an estimated 65 days at sea, although decomposed, remained largely intact, with some adipocere formation [45]. Therefore, whether a body will decompose or be rapidly scavenged may relate, in part, to its position in the water column. The human cases studied in Puget Sound, Monterey Bay and Italy were bodies recovered floating or washed ashore [19, 21, 45] and the pig carcasses in Howe Sound were at depths that allowed bloat, and a later false bloat (caused by gases remaining trapped in organs such as the gut), therefore the carcasses were raised up above the seafloor [23].

The carcasses in the experiments reported here did not go into a bloat stage as the pressure at these depths would prevent bloat from forming [22]. Therefore, they remained in contact with the seabed for the duration of the experiment. Their constant contact with the seabed may have had an impact on much of the faunal colonization and the differences observed between floating carcasses and those on the seabed, thereby impacting the types of scavengers that could access the carcasses.

### Impact of Season

The rate of lyssianassid amphipod arrival on the carcasses and their subsequent scavenging was very similar in both spring and fall and in both caged and exposed carcasses, occurring within minutes of deployment, despite shark depredation in spring. Their skeletonization of the carcasses proceeded very similarly in both seasons, although the amphipods completely covered the cage bars in spring but only reached the first crossbars in fall. Despite this, the fall carcasses were actually skeletonized faster (on Day 3) than spring carcasses which skeletonized one day later. In both seasons, they completely removed all the soft tissue and eventually skin and cartilage, disarticulated the skeleton and moved the skeletal elements out of the anatomically correct position and moved them all around the cage area. Their activity caused bioturbation of the surrounding substrate to a depth of several centimeters and brought heart urchin tests and other detritus to the seafloor surface.

*Pandalus platyceros* were attracted immediately to the preliminary carcass and to the spring carcasses but not to the fall carcasses until Day 7 when very few amphipods still remained, although slender shrimp, *H*. *cf*. *tenuissimus*, did roost on the cage bars periodically from Day 1. However, numbers of *P*. *platyceros* gradually declined on the spring carcasses after skeletonization but dramatically increased in the fall deployment, with very large numbers remaining with the bones until recovery. This may have been impacted by the heavy layer of silt which covered the spring bones as *P*. *platyceros* generally prefers a rocky substrate [46] so may have been repelled once the bones were covered. The fall bones were never covered in silt. In spring, when *P*. *platyceros* arrived immediately after deployment, they appeared to be repelled repeatedly by the encroaching amphipods, feeding only in areas with fewer amphipods and only very rarely being seen in among them. Eventually they were driven from the spring carcasses until the amphipods receded, when they returned. They were not seen on the fall carcasses until the amphipods had receded. The depth of these carcasses was within the range of *P*. *platyceros* (0-500m) although their preference is for 70–90 m [46], but clearly the value of the resource outweighed other issues. Although the physical presence of the amphipods clearly seemed to repel the shrimp, if the activity of the amphipods did reduce oxygen levels as indicated from the fall optode data, then the shrimp may also have been repelled by the low oxygen conditions and only returned when the amphipods receded and the oxygen levels returned to normal.

Relatively few crabs were attracted in either season, in contrast with earlier studies in Saanich Inlet, in which *M*. *magister* removed a large amount of the biomass [24]. It is possible the large numbers of amphipods also repelled other crustaceans and once they receded, very little nutrition remained. As well, almost no fish were seen, especially during the periods of amphipod activity. Lyssianassid amphipods have been reported to drive fish away from carrion [18]. The shortspine thornyhead, *S*. *alascanus*, was often seen to hover in the cage areas for lengthy periods of time, primarily in spring, once the amphipods had receded, but was not observed feeding.

### Impact of Abiotic Parameters

Oxygen levels were, for the most part, at optimum levels for marine animals, being above 2mL/L and higher for the majority of the time [47]. Dissolved oxygen levels dropped dramatically in fall to very low levels for two days during the presence of very large numbers of amphipods. These hypoxic levels cause extremely high levels of stress to most marine animals [47] and may be responsible, in part, for the dearth of other fauna present during the amphipod colonization. In general, oxygen levels were higher in spring coinciding with the increased fresh water in-flows from surface run-off and rain, which added highly oxygenated water.

Temperature in spring was very slightly cooler overall due to the increased levels of cold fresh water run-off which results in slightly cooler water temperatures in spring and also lower conductivity, salinity and density [26]. *Pandalus platyceros* inhabits a much narrower range of temperatures and salinity than others in its genus, preferring temperatures of 7.5–11^°^C and salinities ranging from 26–31 psu [46]. However, temperatures remained optimum throughout the studies, and is rarely limiting for the lyssianassid amphipods recovered here, as they can survive in very low temperatures. In McMurdo Sound, in the Ross Sea in Antarctica, large pieces of seal meat in fish traps were entirely consumed by *Orchomene* and *Orchomenella* spp. in less than 24 h after placement and traps recovered very large numbers of these animals, despite very low temperatures ranging from -2.8 to -0.8^°^C. Fish caught in the traps were frequently eaten alive [18, 48]. So low temperature seems to rarely limit amphipod activity.

## Conclusions

This is the first study to document carcass taphonomy in the open, well-oxygenated waters of the Strait of Georgia and it has demonstrated a dramatically different scavenging progression from that seen earlier in nearby waters.

Earlier studies indicated that a carcass approximating a human body in torso size, skin type and internal bacteria, would be likely to survive for weeks or months, depending on oxygen levels, season, depth and whether it remained in contact with the seabed. However, these studies have shown that in highly oxygenated deeper waters, it can be expected that such a body would be skeletonized in less than four days, although bones could be recovered for six months or more. Related to this observation is the importance of using proxies that approximate human size, rather than relying on whale carcass decomposition, which has a far extended decomposition trajectory.

These observations are also important for recovery divers so that they know what to expect and what to be searching for. It is valuable for recovery divers to be aware of the types of water conditions that are conducive to large amphipod masses as personal conversations with divers who recover human bodies indicate that amphipods are extremely irritating in close proximity, and can even cause a severe panic reaction and pose a drowning risk [22]. It also means that family members can be prepared for whether it is likely an intact or partially intact body will be recovered or whether only bones can be expected.

When bodies or body parts are recovered, such information may also be valuable in estimating a minimum submergence time and indicating types of waters or habitats to which the remains may have been exposed.

Studying marine taphonomy is extremely difficult as the environment is inaccessible and hazardous to divers. VENUS/ONC has provided a unique and extraordinary opportunity to conduct marine carrion experiments in a deep sea context. These experiments are ongoing with different habitats and depths being studied (www.oceannetworks.ca).

## Supporting Information

S1 Video Six-gill shark (*Hexanchus griseus* (Bonnaterre, 1788)) feeding on an openly exposed pig carcass in the Strait of Georgia at 300m eight hours after deployment (Ocean Network Canada’s VENUS observatory). (WMV)

S2 Video Lyssianassid amphipods settling on both the carcasses but a six-gill shark (*Hexanchus griseus* (Bonnaterre, 1788)) swims over the carcass platform in spring, six hours after deployment. It is unable to access the caged carcass but begins to feed on the exposed carcass (Ocean Network Canada’s VENUS observatory).
(WMV)

S3 Video A giant pacific octopus (*Enteroctopus dofleini* (Wülker, 1910)) exploring the spring cage and bones on Day 9 (Ocean Network Canada’s VENUS observatory). (WMV)
